# Combined Intrinsic Local Functional Connectivity With Multivariate Pattern Analysis to Identify Depressed Essential Tremor

**DOI:** 10.3389/fneur.2022.847650

**Published:** 2022-05-10

**Authors:** Xueyan Zhang, Li Tao, Huiyue Chen, Xiaoyu Zhang, Hansheng Wang, Wanlin He, Qin Li, Fajin Lv, Tianyou Luo, Jin Luo, Yun Man, Zheng Xiao, Jun Cao, Weidong Fang

**Affiliations:** ^1^Department of Radiology, The First Affiliated Hospital of Chongqing Medical University, Chongqing, China; ^2^Department of Neurology, The First Affiliated Hospital of Chongqing Medical University, Chongqing, China; ^3^Department of Psychiatry, The First Affiliated Hospital of Chongqing Medical University, Chongqing, China

**Keywords:** essential tremor, depression, regional homogeneity, resting-state functional magnetic resonance imaging, machine learning

## Abstract

**Background:**

Although depression is one of the most common neuropsychiatric symptoms in essential tremor (ET), the diagnosis biomarker and intrinsic brain activity remain unclear. We aimed to combine multivariate pattern analysis (MVPA) with local brain functional connectivity to identify depressed ET.

**Methods:**

Based on individual voxel-level local brain functional connectivity (regional homogeneity, ReHo) mapping from 41 depressed ET, 43 non-depressed ET, and 45 healthy controls (HCs), the binary support vector machine (BSVM) and multiclass Gaussian Process Classification (MGPC) algorithms were used to identify depressed ET patients from non-depressed ET and HCs, the accuracy and permutations test were used to assess the classification performance.

**Results:**

The MGPC algorithm was able to classify the three groups (depressed ET, non-depressed ET, and HCs) with a total accuracy of 84.5%. The BSVM algorithm achieved a better classification performance with total accuracy of 90.7, 88.64, and 90.48% for depressed ET vs. HCs, non-depressed ET vs. HCs, and depressed ET vs. non-depressed ET, and the sensitivity for them at 80.49, 76.64, and 80.49%, respectively. The significant discriminative features of depressed ET vs. HCs were primarily located in the cerebellar-motor-prefrontal gyrus-anterior cingulate cortex pathway, and for depressed ET vs. non-depressed ET located in the cerebellar-prefrontal gyrus-anterior cingulate cortex circuits. The partial correlation showed that the ReHo values in the bilateral middle prefrontal gyrus (positive) and the bilateral cerebellum XI (negative) were significantly correlated with clinical depression severity.

**Conclusion:**

Our findings suggested that combined individual ReHo maps with MVPA not only could be used to identify depressed ET but also help to reveal the intrinsic brain activity changes and further act as the potential diagnosis biomarker in depressed ET patients.

## Introduction

Depression is one of the most common non-motor disorders in essential tremor (ET) patients, and approximately 25–50% of the ET patients have mild to severe depressive symptoms. Growing evidence (1–4) suggested that depression may be a pre-motor marker for the development of ET, and depressive symptoms in ET may reflect the disease process itself. The 2018 consensus statement of the Movement Disorder Society redefined ET with depression as a new entity “ET plus” (5). However, the new diagnosis criterion of ET plus is only based on clinical features, and it is still unclear whether ET with depression is associated with underlying identifiable brain activity changes, especially the absence of the objective diagnosis biomarker.

Resting-state functional magnetic resonance imaging (rs-fMRI) is a non-invasive technology based on blood-oxygen-level-dependent (BOLD) (6), and it has been considered one of the most promising approaches for revealing the intrinsic brain activity changes and further establishing diagnosis biomarkers. Using local brain connectivity (7), seed-based brain connectivity (8), and independent component analysis (9) of rs-fMRI, our previous studies had demonstrated that the cerebello-thalamo-cortical pathway dysfunction is associated with tremor and cognitive impairment in ET patients. Among the above rs-fMRI metrics, regional homogeneity (ReHo) is one of the most used indicators, and it investigated brain local functional connectivity by calculating the temporal correlations between a voxel and its adjacent voxels without any prior knowledge. More recently, we revealed that the ReHo changes in the frontal-cerebellar-anterior cingulate cortices pathway were related to depressed ET patients (10). However, due to traditionally mass-univariate analyses, all these above studies could not be used to diagnose the individual depressed ET patient, and had difficulty sensitively identifying the diagnosis biomarker. Multivariate pattern analysis (MVPA) is a new method based on machine learning algorithm, which has been widely used to analyze the spatial pattern information for fMRI classification with good generalization ability (11). Owing to multivariate properties, the MVPA can achieve greater sensitivity for discovering voxel-level subtle and spatially distributed changes of intrinsic brain activity, and are sensitive enough to perform classification at the single-subject level. Combining global brain connectivity of rs-fMRI with MVPA (12), our latest studies showed good classification performance to identify depressed ET patients. However, whether the MVPA based on local brain connectivity can realize automatic identification of depressed ET has not previously been investigated.

In this study, we combined voxel-level local brain connectivity (regional homogeneity, ReHo) mapping of rs-fMRI with MVPA (multiclass Gaussian Process Classification and binary support vector machine algorithms) to identify depressed ET patients from non-depressed ET and healthy controls (HCs). We expected that these classification models could achieve good accuracy and the selected significant discriminative brain region features would help to establish potential diagnostic biomarkers and reveal the intrinsic brain activity pathogenesis in depressed ET patients.

## Methods and Materials

### Participants

Depressed ET patients and non-depressed ET patients were recruited at the movement disorders or psychiatry outpatient clinic of the First Affiliated Hospital of Chongqing Medical University (Chongqing, China). Healthy controls were recruited from the local individuals by poster advertisements, and HCs reported having a first-degree or second-degree relative with ET or Parkinson's diseases were discarded. The detailed inclusion criteria for all subjects and head motion control are described in Supplementary Materials. After controlling for image quality and head motion, 3 depressed ET patients, 2 non-depressed ET patients, and 3 HCs with FDpower head motion > 0.2 mm were removed from our study. Eventually, 41 depressed ET, 43 non-depressed ET, and 45 age- and sex-matched HCs were included in our study.

The depression severity of each subject was evaluated by the 17-item Hamilton Depression Rating Scale (HDRS-17) (13), and all patients with a score of at least 7 points were considered depressive. The Hamilton Anxiety Rating Scale (HARS-14) (14) assessed the anxiety severity of all participants. Tremor severity was assessed with the Fahn-Tolosa-Marin Tremor Rating Scale (FTM-TRS). Meantime, to consider a ceiling effect for severe tremor while tremor amplitude > 4 cm for the TRS scale, the Essential Tremor Rating Assessment Scale (TETRAS) (15) was also adopted to assess tremor severity in our study. The MMSE was used to briefly assess cognitive function and screen for dementia.

### MRI Acquisition

Resting-state fMRI images, 3D T1-weighted images, and T2-FLAIR images were acquired using a GE Signa Hdxt 3T scanner (General Electric Medical Systems, Milwaukee, WI, United States). Rubber earplugs were used to reduce noise, and foam cushioning was used to reduce motion artifacts. During the scan, participants were instructed to keep their eyes closed and stay awake (which was confirmed *via* intercom immediately after the rs-fMRI scan). Resting-state fMRI images were obtained using a standard echo-planar imaging (EPI) pulse sequence with the following parameters: 33 axial slices, slice thickness/gap = 4.0/0 mm, matrix = 64 × 64, TR = 2000 ms, TE = 40 ms, flip angle = 90°, FOV = 240 × 240 mm, and a total of 240 volumes were obtained (duration = 8 min). High-resolution 3D T1-weighted images (TR = 8.3 ms, TE = 3.3 ms, flip angle = 15°, slice thickness/gap = 1.0/0 mm, FOV = 240 × 240 mm, and matrix = 256 × 192) and T2-weighted FLAIR images (TR = 8,000 ms, TE = 126 ms, TI = 1,500 ms, slice thickness/gap = 5.0/1.5 mm, FOV = 240 × 240 mm, and matrix = 256 × 192) were also acquired. We did not use the T2-weighted FLAIR images for data processing, but they were used for image evaluation.

### Image Preprocessing

Functional imaging preprocessing was performed using statistical parametric mapping software (SPM12; www.fil.ion.ucl.ac.uk/spm) and the Data Processing Assistant for Resting-State fMRI software (DPARSF; http://rfmri.org/DPARSF). The first 10 volumes of the functional images were discarded for the signal equilibrium and participants' adaptation to the scanning noise and only the remaining 230 images were processed and analyzed. The main steps of image preprocessing were as follows: (1) Slice-timing correction was performed to correct the layer time to eliminate the error caused by the removal of sequences at the beginning of the scan; (2) realignment was performed to correct the differences in head translation or rotation during data collection, and resulting in Friston 24 head motion parameters. These parameters were employed to assess head movement and ensure the quality of rs-fMRI data; (3) the functional images were normalized to the standard Montreal Neurological Institute (MNI) space by Diffeomorphic Anatomical Registration Through Exponentiated Lie algebra (DARTEL) and resampled to 3 × 3 × 3 mm^3^; (4) the T1 images were co-registered to the mean rs-fMRI data for each subject. Specifically, T1 images were segmented into gray matter (GM), white matter (WM), and cerebrospinal fluid (CSF). All the GM, WM, and CSF images were resampled to 1.5 × 1.5 × 1.5 mm^3^ and spatially normalized to the MNI space using both affine transformation and non-linear deformation, after that, resampled to 3 × 3 × 3 mm^3^ voxel resolution with rs-fMRI and the deformation field was applied to the rs-fMRI data; (5) the nuisance covariates including the Friston 24 head motion parameters, white matter signal, cerebrospinal fluid signal, and the mean time series of the whole brain were regressed out; (6) for detrending, we used 1st order polynomial functions to remove the linear trend produced by a rise in temperature due to the machine working or adaptation of the subject over time; (7) the resting-state BOLD signal was filtered using a filter with a frequency band of 0.01–0.08 to decrease low-frequency drift and high-frequency noise. To ensure that neighboring regional BOLD time courses did not show a spurious increase in connectivity strength, smoothing was performed after calculating the individual ReHo value, and the flow chart of imaging preprocessing is presented in Supplementary Figure S1.

### Calculation of Voxel-Level Local Functional Connectivity Mapping

In this study, we used ReHo to describe the voxel-level local functional connectivity. Each individual ReHo map was generated by calculating Kendall's coefficient of concordance (KCC) of the time series of a voxel with those of 26 nearest neighbors. The KCC was calculated by the following formula:


(1) $$\begin{array}{r} W\;=\;\frac{\sum {\left(R_{i}\right)}^{2}-n{\left(\bar{R}\right)}^{2}}{\frac{1}{12}K^{2}\left(n^{3}-n\right)} \end{array}$$

Where *W* is the KCC among given voxels, ranging from 0 to 1; *R*_*i*_ is the sum rank of the *i*th time point; $\bar{R}=\left(\left(n+1\right)K\right)/2\;$is the mean of the *R*_*i*_'s; *K* is the number of time series within a measured cluster (*K* = 27, one given voxel plus the number of its neighbors) and *n* is the number of ranks (16). Then a whole-brain mask provided by DPARSF was used to remove non-brain areas. To reduce the influence of individual variations, each ReHo map was divided by the global mean ReHo of each participant. Ultimately, the standardized ReHo maps were smoothed with a Gaussian kernel of 4 mm full-width at half-maximum (FWHM) for noise reduction. All the subsequent analyses were carried out based on smoothed ReHo maps (smReHo).

### Multivariate Pattern Machine Learning Classification Analysis

The Pattern Recognition for Neuroimaging Toolbox (PRoNTo version 2.0; http://www.mlnl.cs.ucl.ac.uk/pronto/) (17) within SPM12 was used to perform multivariate pattern machine learning (MVPA) classification analysis. The machine library in PRoNTo included four classification algorithms: support vector machine (SVM), binary and multiclass Gaussian Process classifier (BGPC and MGPC), and L1-multiple kernel learning. To solve the three-class (depressed ET, non-depressed ET, and HCs) and two-class (depressed ET vs. non-depressed ET, depressed ET vs. HCs, and non-depressed ET vs. HCs) classification problems, the MGPC and Simple L1-Multiple Kernel Learning (Simple-MKL) algorithms were used to deal with three-class classification, and linear SVM and BGPC algorithms were adopted to tackle two-class classification. Briefly, the MVPA approach was composed of five main analysis modules: dataset specification, feature set selection, model specification, model estimation, and weights computation. In the dataset specification and feature set selection, individual participants' ReHo maps served as inputs features for the machine learning algorithms, in which a resting-state design was modeled with no conditions, and the DARTEL gray matter mask was applied. A feature set was prepared on whole-brain voxel-level smReHo data. In the model specification and model estimation, the features were mean-centered, and MGPC, Simple-MKL, binary SVM, and BGPC algorithms were used to test whether the individual smReHo maps could be used to discriminate these subjects. These subjects were divided into training and testing sets and a leave-one-subject-out cross-validation (LOSOCV) was used. During the training phase, a learning algorithm was trained with the original training set, and during the test phase, the trained learning algorithm was applied to predict the labels from the unseen samples on the testing set. For example, in the linear case, the learned function relied on a linear combination of the feature vectors x_i_, i.e., $f\left(x_{i}\right)=w_{0}+w^{T}x_{i}$. The weights *w* ∈ R^d^ represented the relative contribution of each feature for the classification model (18). The area under the receiver operating characteristic curve (AUC), receiver operating characteristic (ROC) curve (only in binary SVM and BGPC), accuracy, sensitivity, specificity, total accuracy, positive predictive value, and negative predictive value were calculated to assess the classification performance. We acquired the prediction labels for every participant, which were used to build the confusion matrix, and we further used permutation testing (1,000) to assess the significance of these models' performance and to locate the significant discriminative features. More specifically, we repeated the permutation cross-validation procedure test 1,000 times and counted how many times the value of these accuracy measures was equal to or higher than the correct one. The *p*-value was then calculated by dividing this number by the number of permutations (1,000). To locate the significant discriminative features, the contribution of each voxel to classification was calculated, and the voxels' *p*-value was calculated by dividing this number by the number of permutations (1,000), and they were projected to generate the discriminative map, and the cluster size > 30 voxels was adopted.

### Statistical Analysis

Statistical analysis was carried out using Statistical Package for the Social Sciences (SPSS) Version 20.0. The demographic and clinical information was analyzed by descriptive statistics and presented as means and deviations. Kolmogorov–Smirnov tests were performed to assess the normality of these data. Among the three groups, qualitative data were assessed using the chi-square test based on the distribution, and quantitative variables were analyzed using a one-way analysis of variance (ANOVA) test. *Post hoc* analysis using Bonferroni correction was performed when appropriate. The *p*-values of 0.05 were considered statistically significant (two-tailed). Then, Pearson's (for normal distribution) or Spearman's (for non-normal distribution) correlation analysis was applied to explore relationships among HDRS-17 scores, age, educational level, age of tremor onset, tremor duration, and scores on TRS parts A and B, TRS part C, TETRAS, TETRAS-ADL, MMSE, and HARS-14 in depressed ET patients.

To further explore whether the significant discriminative features could be used to explain depression severity in depressed ET patients, the significant discriminative features clusters (BSVM: depressed ET vs. HCs) were defined as the region of interest (ROI). Briefly, we extracted smReHo values from these ROIs, and a partial Pearson's correlation analysis between smReHo values and the HDRS-17 scores of the depressed ET patients was performed with a Bonferroni multiple comparison correction, controlling for age, sex, years of education, and scores of TRS parts A and B, TRS part C, MMSE, and HARS-14, and head motion FDpower as covariates.

## Results

### Demographic and Clinical Characteristics

Demographic and clinical information are shown in Table 1, and the age, education level, tremor of onset, and scores on TRS parts A and B, TRS part C, MMSE, HDRS-17, and HARS-14 in depressed ET patients show a normal distribution (*p* = 0.63, 0.12, 0.32, 0.66, 0.33, 0.06, 0.97, and 0.06, respectively), and the tremor duration in the depressed ET patients shows a non-normal distribution (*p* = 0.035). There is no significant correlation among these clinical data.

**Table 1:** Demographic and clinical features of depressed ET, non-depressed ET, and HCs.

**Measure**	**DET (41)**	**ET (43)**	**HCs (45)**	**Statistic**	***p*** **-value**		
					**DET vs. HCs**	**ET vs. HCs**	**DET vs. ET**
**Demographic**
Age (year)	47.85 ± 15.66	44.49 ± 13.73	46.67 ± 13.16	F = 0.99	0.48	0.47	0.16
Sex (male/female)	26:15	28:15	28:17	x^2^ = 0.79	0.91	0.78	0.87
Education (year)	14.73 ± 4.17	12.40 ± 3.77	12.22 ± 3.42	F = 5.75	0.03	0.83	0.06
Handedness (R/L)	41:0	49:0	43:0	x^2^ = 0	1	1	1
**Clinical of psychology and cognitive**
HDRS-17	18.98 ± 6.35	3.49 ± 1.56	2.11 ± 1.17	F = 261.56	1e^−6^	0.09	1e^−6^
MMSE	26.00 ± 1.40	26.33 ± 1.51	28.67 ± 1.35	F = 46.08	1e^−6^	1e^−6^	0.30
HARS-14	7.56 ± 3.47	4.09 ± 1.66	2.04 ± 1.17	F = 63.47	1e^−6^	0.5e^−5^	1e^−6^
**Clinical of tremor**
Tremor of onset	34.93 ± 11.84	33.09 ± 10.87	NA	T = 0.74	NA	NA	0.46
Tremor duration	13.90 ± 9.40	11.42 ± 6.82	NA	T = 1.39	NA	NA	0.17
TRS-parts A&B	22.66 ± 7.28	20.93 ± 7.77	NA	T = 1.05	NA	NA	0.30
TRS-part C	14.05 ± 4.74	10.70 ± 5.22	NA	T = 3.08	NA	NA	0.03
TETRAS	18.32 ± 8.27	17.41 ± 6.79	NA	T = 0.57	NA	NA	0.57
TETRAS-ADL	21.39 ± 6.51	20.53 ± 5.19	NA	T = 2.37	NA	NA	0.09

*DET, depressed ET; ET, essential tremor; HCs, healthy controls; HDRS-17, 17-item Hamilton Depression Rating Scale; MMSE, Mini-Mental State Examination; HARS-14, 14-item Hamilton Anxiety Rating Scale; TRS, Fahn-Tolosa-Marin Tremor Rating Scale; TETRAS, Essential Tremor Rating Assessment Scale; TETRAS-ADL, Essential Tremor Rating Assessment Scale-Activities of Daily Living; N/A, not applicable*.

### Classification Performances

The MGPC algorithm achieved a good classification performance with a total accuracy of 84.5%, and sensitivity for depressed ET, non-depressed ET, and HCs at 80.49, 72.09, and 100.00%, respectively. The Simple-MKL algorithm achieved an overall classification accuracy of 79.07%, and sensitivity for depressed ET, non-depressed ET, and HCs at 82.93, 65.12, and 88.89%, respectively. For all performance evaluation metrics except the sensitivity for depressed ET patients, the Simple-MKL algorithm performed worse than the multiclass Gaussian Process Classification algorithm. Supplementary Figures S2, S3 separately show the confusion matrixes and classification performances of MGPC and Simple-MKL algorithms for classifying depressed ET, non-depressed ET, and HCs.

**Figure 1:**
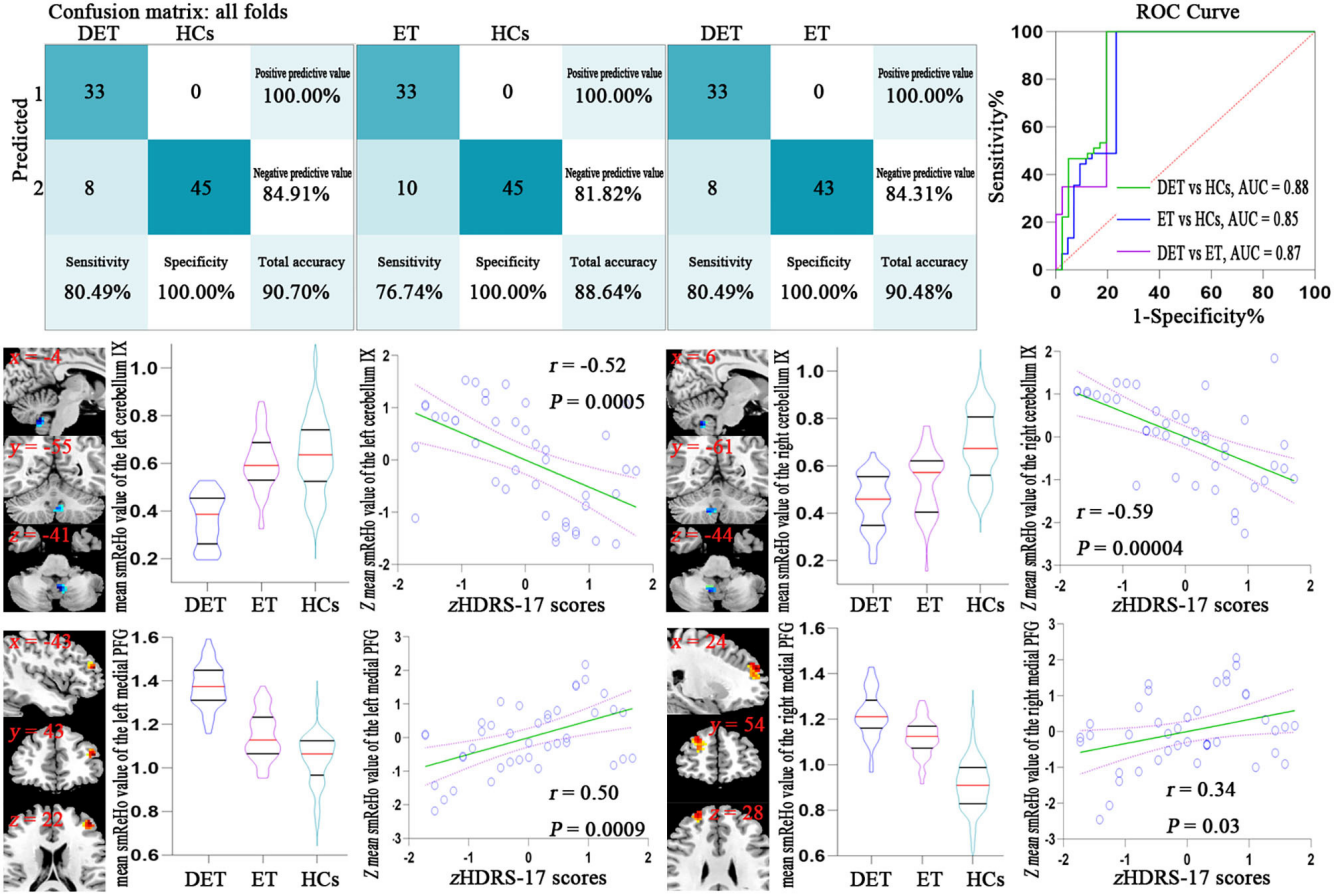
Confusion matrix and ROC curve of the binary SVM algorithm (upper pictures). Correlation analysis results between the smReHo values of significant discriminative features and the HDRS-17 scores in depressed ET patients (lower pictures). Bonferroni multiple comparison corrections, corrected *p* < 0.05/18*(18-1)/2. smReHo, smoothed regional homogeneity; PFG, prefrontal gyrus; zHDRS-17 scores, z-transformed Hamilton Depression Rating Scale 17-item scores; DET, depressed essential tremor; ET, non-depressed essential tremor; HCs, healthy controls; ROC, receiver operating characteristic; AUC, area under the curve.

Figure 1 (upper pictures) shows the confusion matrix and classification performances of SVM of depressed ET vs. HCs, non-depressed ET vs. HCs, and depressed ET vs. non-depressed ET. These SVM classifications could reach a good classification performance with accuracy at 90.70, 88.64, and 90.48% respectively, and the permutation test with statistically significant balanced accuracy, sensitivity, and specificity at *p* < 0.001. The classification performances of BGPC were worse than that of SVM, and the confusion matrixes and classification performances of BGPC are shown in the Supplemental Material.

**Figure 2:**
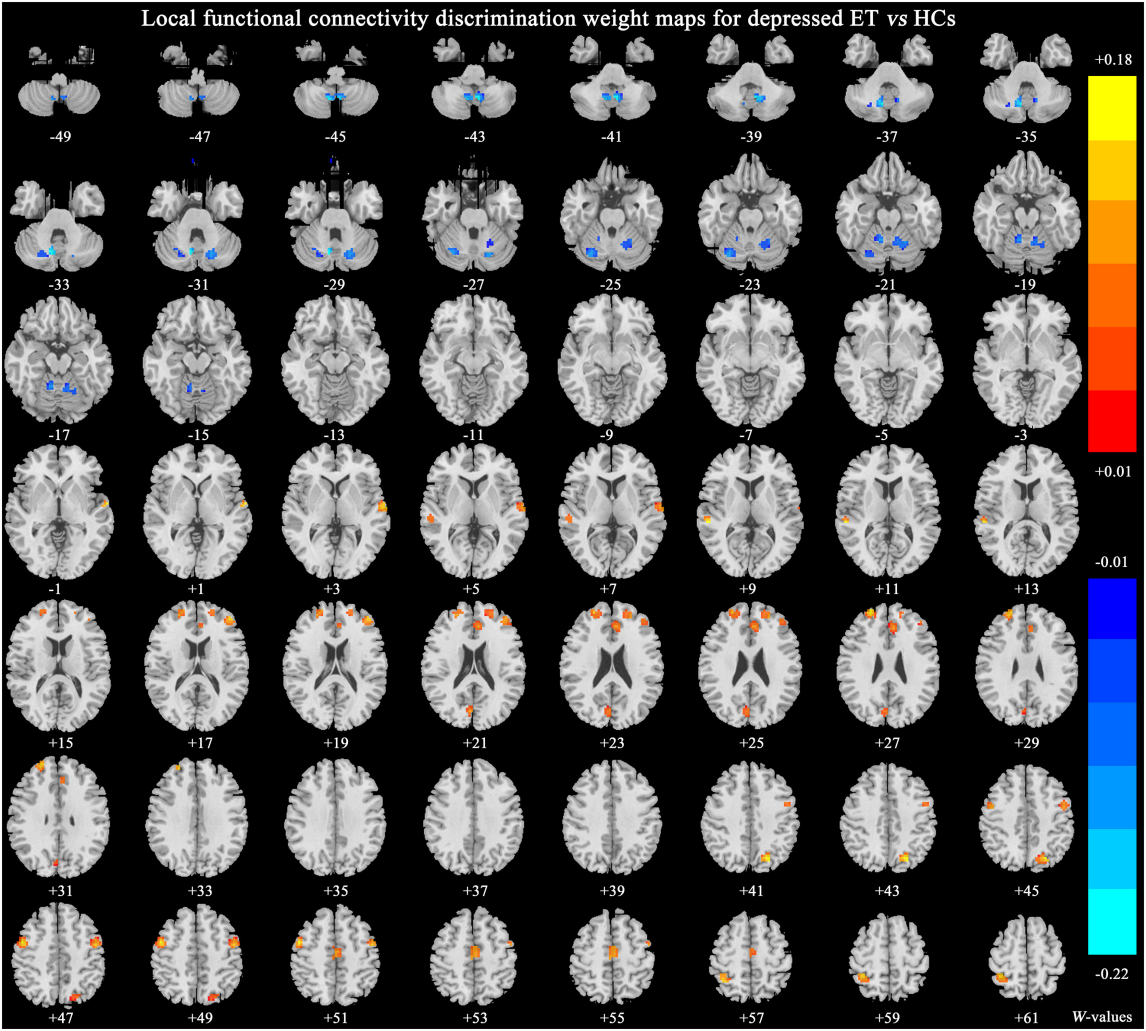
The brain regions of significant discriminative features in the classification of depressed ET vs. HCs. ET, essential tremor; HCs, healthy controls.

Figure 2 shows significant discriminative features of depressed ET vs. HCs with permutation test at *p* < 0.001. Positive (mean depressed ET > HCs) discriminative features are located in the bilateral supplementary motor cortices, bilateral precentral cortices, bilateral anterior cingulate cortices, bilateral precuneus gyri, bilateral middle and superior prefrontal gyri, bilateral inferior parietal lobules and bilateral cuneus, and negative (mean depressed ET < HCs) discriminative features are located in bilateral cerebellum lobules IV~V, bilateral cerebellum lobules VI, bilateral cerebellum lobules VIII, bilateral cerebellum lobules Crus 1, and bilateral cerebellum lobules IX.

**Figure 3:**
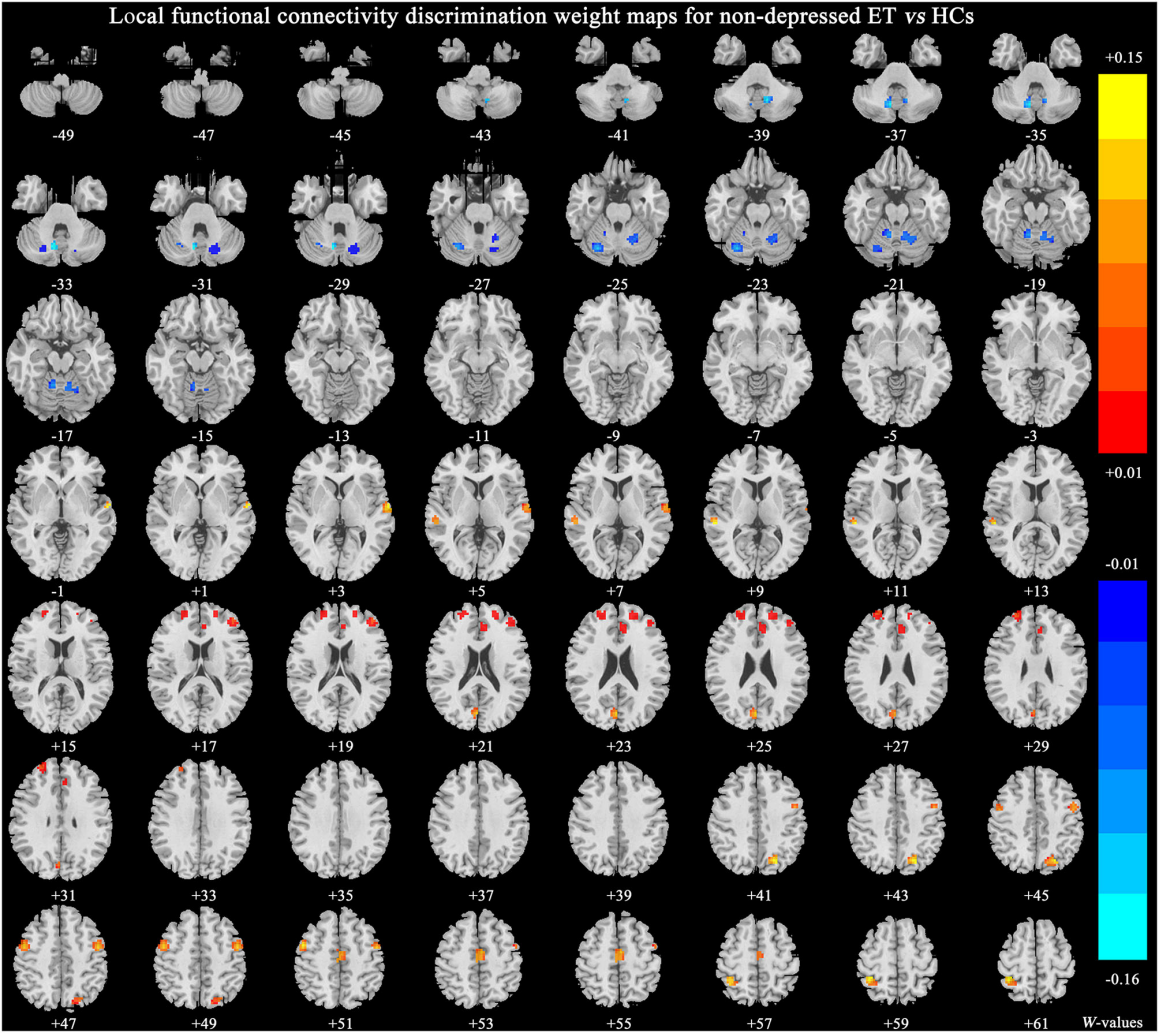
The brain regions of significant discriminative features in the classification of non-depressed ET vs. HCs. ET, essential tremor; HCs, healthy controls.

Figure 3 shows significant discriminative features of non-depressed ET vs. HCs with permutation test at *p* < 0.001. The significant discriminative features were similar to that of depressed ET vs. HCs, except the negative features in bilateral cerebellum lobules IX were not revealed.

**Figure 4:**
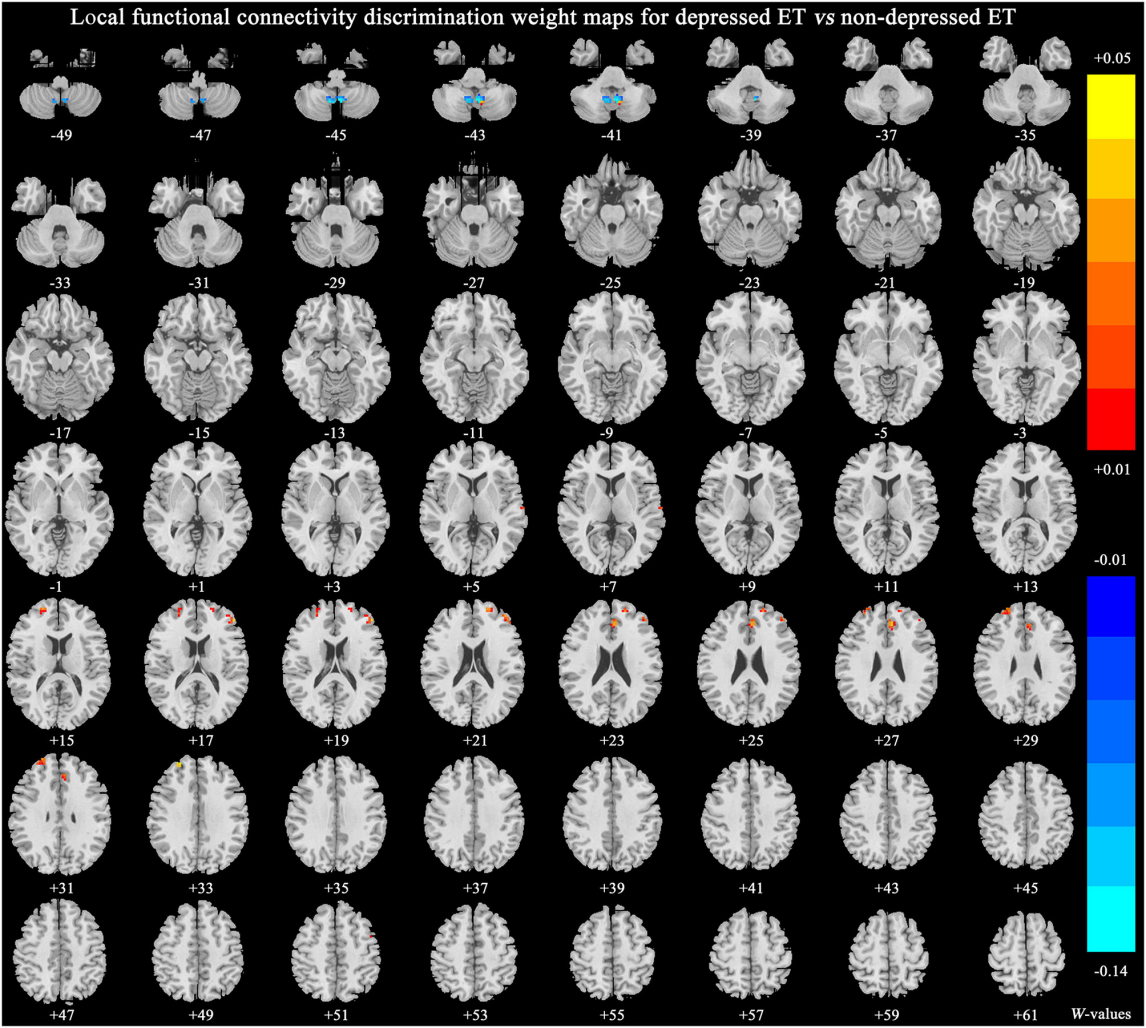
The brain regions of significant discriminative features in the classification of depressed ET vs. non-depressed ET. ET, essential tremor; HCs, healthy controls.

Figure 4 shows significant discriminative features of depressed ET vs. non-depressed ET with permutation test at *p* < 0.001. Positive (mean depressed ET > non-depressed ET) discriminative features are located in the bilateral middle and superior prefrontal gyri and bilateral anterior cingulate cortices, and negative (mean depressed ET < non-depressed ET) discriminative features are located in bilateral cerebellum lobules IX.

The brain regions and peak MNI coordinates of significant discriminative features in the classification of binary SVM are listed in Supplementary Table S1.

### Partial Pearson's Correlation Analysis

A total of eighteen clusters of the significant discriminative features of depressed ET vs. HCs were revealed and all of the 18 clusters were defined as 18 ROIs, and the smReHo values of these ROIs were abstracted. Figure 1 (lower pictures) shows the partial Pearson's correlation analysis results, and the significant correlation between HDRS-17 scores and smReHo values of the left middle prefrontal gyrus (positive), and bilateral cerebellum lobules IX (negative) and right middle and superior prefrontal gyri (marginal positive) were found in depressed ET patients.

## Discussion

To our knowledge, this is the first study to combine local brain functional connectivity with a multivariate pattern machine learning approach to identify depressed ET patients from non-depressed ET and HCs, and three main findings were gained: (1) both the MGPC and SVM could achieve good classification performance, especially for HCs with sensitivity at 100%; (2) the significant discriminative features were mostly located in cerebellar-motor-prefrontal gyri-anterior cingulate cortices pathway; (3) the significant discriminative features in bilateral cerebellum lobules IX and prefrontal gyri could be used to explain depression severity in depressed ET patients.

### Multivariate Pattern Analysis in Essential Tremor

The typical analysis in neuroimaging was based on mass-univariate analysis, and it assumed that the activity of one brain region is independent of the other brain regions. Just as our recent research revealed that changes of ReHo in frontal-cerebellar-anterior cingulate cortex circuits were associated with depressed ET (10). Although the mass-univariate approach had powerful insights over the years, due to the voxel interaction, high-dimensional and multivoxel properties of the rs-fMRI data, these results could not be used to diagnose the individual depressed ET patients, and it was not sensitive to reveal the subtle and spatially distribution changes of these rs-fMRI metrics including ReHo. Recently, the adoption of MVPA could not only consider the above properties of the rs-fMRI data but also extract stable and identifiable features from rs-fMRI data to distinguish subjects at the individual level (19). Previously, the neural networks machine learning model classified Parkinson's disease and ET based on balance and gait characteristics and achieved an accuracy of 89% (17). Voice analysis with a support vector machine classifier objectively discriminated between healthy controls and ET patients who did and did not manifest clinically overt voice tremors with the accuracy of 97.1 and 97.9% (20). However, these clinical behavioral symptoms were less stable, direct, and accurate than neuroimaging data. In our study, we adopted local brain functional connectivity of rs-fMRI as input features to identify depressed ET. Finally, the MGPC model was able to classify the three groups (depressed ET, non-depressed ET, and HCs) with total accuracy of 84.5% and the BSVM model achieved a better classification performance with total accuracy of 90.7, 88.64, and 90.48% for depressed ET vs. HCs, non-depressed ET vs. HCs, and depressed ET vs. non-depressed ET, and with sensitivity of 80.49, 76.64, and 80.49%, respectively. The good classification performance of MGPC and binary SVM suggested that these classification algorithms not only provide a potential chance for diagnosing the individual depressed ET patients but also help to reveal the spatial distribution changes of local brain connectivity in depressed ET patients.

### The Cerebellar-Motor-Prefrontal Gyrus-Anterior Cingulate Cortex Pathway Associated With Depression in ET Patients

Amounting evidence has shown that the prefrontal cortex is responsible for top-down mood and attention regulation (21). This area plays an important role in the pathogenesis of primary depression and movement disorders with depression such as Parkinson's disease (22, 23). In the past few years, a major depressive disorder model mentioned the importance of the ventromedial prefrontal cortex, anterior cingulate cortex, and lateral parietal cortex, which might be related to self-referential problems and negative ruminations (24). The anterior cingulate cortex which consists of affective and cognitive subdivisions plays an important role in regulating cognitive and emotional functions and may be involved in the pathophysiology of major depression (25). Furthermore, the anterior cingulate cortex is not only an important hub of the default mode network but also a key area of the limbic system. The default mode network has been generally linked to self-referential processing, affective cognition, and emotion regulation (26), and disturbances of the default mode network have been confirmed in many neurological and psychiatric disorders including ET (27). Using voxel-wise global brain connectivity mapping combined with MVPA, a study conducted by our team has shown that the major associated discriminative features in depressed ET were mainly located in the cerebellar-motor-prefrontal cortex circuits (12). Compared to the previous study, the present study was the first to combine local brain functional connectivity with the MVPA approach to identify depressed ET patients from non-depressed ET and HCs which may help deepen our understanding of the depression pathogenesis of ET from different perspectives. Similar to the studies mentioned above, our study confirmed the involvement of the prefrontal cortex, anterior cingulate cortex, and cerebellum among the discriminative features of depressed ET vs. non-depressed ET, but also brought additional highlights. It was worth noting that these areas were mainly located in the cerebellar-prefrontal gyrus-anterior cingulate cortex circuits. Understandably, we found the involvement of the motor cortex in depressed ET in contrast to HCs. Consequently, the significant discriminative features of depressed ET vs. HCs were mainly located in the cerebellar-motor-prefrontal gyrus-anterior cingulate cortex pathway. As compared to non-depressed ET and HCs, depressed ET showed increased ReHo in the bilateral middle and superior prefrontal gyrus and bilateral anterior cingulate cortex, whereas decreased ReHo in the bilateral cerebellum IX. We also found that no significant correlation existed between HDRS-17 scores and the tremor severity or tremor duration. This result was consistent with a previous study by a US research team suggesting that depression may be a separable construct in depressed ET (28), which could dictate how the depressed ET patient copes with his/her depressive symptoms. To some extent, this reflected that depression in ET may be associated with identifiable underlying brain changes, rather than a secondary psychiatric response to disabling tremors.

Many studies have shown that functional abnormalities of the cerebellum have been a consistent finding in ET (29). Abnormalities in the cerebellar-thalamo-cortical network of ET patients have been extensively reported in previous studies (30). In terms of neuropsychiatric symptoms, several neuroimaging studies revealed that affective disturbances such as anxiety and depression have been related to the cerebellum in the context of the cerebellar cognitive affective syndrome (CCAS) (31). And abnormalities in the cerebellum in association with depression have been consistently found, indicating the involvement of cerebellar dysfunction in depressive disorders (32). Indeed, the existing study has reported that cortical-cerebellar circuits were linked to non-motor symptoms in ET (33). In addition, partial correlation analysis in this study showed that depressive symptoms was independent of tremor severity. The above findings suggest that the cerebellum not only plays an important role in the generation of tremors but also is involved in depressive symptoms in ET.

Previous postmortem observations demonstrated that only cerebellum (e.g., increased numbers of torpedoes and loss of Purkinje cells) and brainstem (e.g., Lewy bodies and depletion of neurons in the locus coeruleus) existed identifiable structural changes in the ET brain (34, 35), and the other brain regions including prefrontal cortices, motor cortices, and anterior cingulate cortices and did not have neuropathology abnormalities. For reasons stated above, we inferred that the cerebellum may pose a key pathogenesis role through the cerebellar-motor-prefrontal gyrus-anterior cingulate cortex pathway involved in depressed ET.

Several limitations of our study are worth mentioning for future improvements. First, in this study, both binary SVM- and MGPC-supervised learning approaches could be used to classify depressed ET, non-depressed ET, and HCs. Although supervised approaches could be preferable than the unsupervised ones when high-quality, representative, and correctly labeled data are available for training, the unsupervised learning algorithms based on a larger follow-up sample size may give perfect classification performance and even achieve a clinical diagnosis state. Second, due to the lack of biological and pathogenic markers, the diagnosis of ET mainly depended on clinical symptoms and neuropsychological assessment. Therefore, all the ET patients included in our study had long follow-up periods supplemented with electromyography results to reduce the incidence of misdiagnosis.

## Conclusion

Our findings suggested that combining the local functional connectivity maps with MGPC and binary SVM algorithms could achieve good classification performance to identify depressed ET patients. The spatially distributed patterns of ReHo changes in the cerebellar-prefrontal gyrus-anterior cingulate cortex circuits not only acted as the significant discriminative features but also helped us to understand the pathogenesis underlying depression in ET patients.

## Data Availability Statement

The raw data supporting the conclusions of this article will be made available by the authors, without undue reservation.

## Ethics Statement

The studies involving human participants were reviewed and approved by Ethics Committee of the First Affiliated Hospital of Chongqing Medical University. The patients/participants provided their written informed consent to participate in this study.

## Author Contributions

XZ conception and execution of the research project, design and execution of statistical analysis, and manuscript preparation in writing of the first draft. LT conception and organization of the research project, statistical analysis, design execution, and manuscript preparation of the review and critique. HC and XZ execution of the research project, statistical analysis, review and critique, and manuscript preparation of the review and critique. HW and WH execution of the research project, statistical analysis, and manuscript preparation of the review and critique. QL and FL execution of the research project, statistical analysis of review and critique, and manuscript preparation of the review and critique. TL and JL execution of the research project and manuscript preparation of the review and critique. YM, ZX, and JC organization of the research project, statistical analysis of the review and critique, and manuscript preparation of the review and critique. WF conception and organization of the research project, design of statistical analysis, execution, review and critique, and manuscript preparation in review and critique. All authors contributed to the article and approved the submitted version.

## Funding

This work was supported by the National Natural Science Foundation of China (NSFC: 81671663) and the Natural Science Foundation of Chongqing (NSFCQ: cstc2014jcyjA10047).

## Conflict of Interest

The authors declare that the research was conducted in the absence of any commercial or financial relationships that could be construed as a potential conflict of interest.

## Publisher's Note

All claims expressed in this article are solely those of the authors and do not necessarily represent those of their affiliated organizations, or those of the publisher, the editors and the reviewers. Any product that may be evaluated in this article, or claim that may be made by its manufacturer, is not guaranteed or endorsed by the publisher.
