# Supplementary material for: Combined Intrinsic Local Functional Connectivity With Multivariate Pattern Analysis to Identify Depressed Essential Tremor
Source: Front Neurol. 2022 May 10;13:847650. doi: 10.3389/fneur.2022.847650 (PMC9127760; doi:10.3389/fneur.2022.847650)
Supplement: Supplementary file 1 [file Data_Sheet_1.docx]

**Combined intrinsic local functional connectivity with multivariate pattern analysis to identify depressed essential tremor**

***Supplementary Material***

1. Supplementary Material
   1. Inclusion criteria for all subjects

The inclusion criteria for all subjects were: 1) all ET patients were diagnosed by two movement specialists based on the 2018 Consensus Criteria of the Movement Disorder Society (1) ; 2) the patients had an onset age between 18 to 55 years old, and patients with earlier or later onset were not included; 3) the patients were not treated with any anti-ET or anti-depressant medications before the baseline fMRI scan (only the baseline fMRI scan data were used in this study); 4) the patients were without any apparent cognitive impairment (Mini-Mental State Examination (MMSE) scores > 24) and were right-handed; 5) the patients presented with moderate or greater amplitude kinetic tremor (tremor rating ≥ 2 during at least three tests); 6) patients were excluded in this study if they complied with the diagnosis of Parkinson’s disease (PD), secondary causes of PD (such as Parkinsonism), dystonia, and tremor of other origins (such as stroke, tumor, and trauma); 7) the depressed ET patients met the Diagnostic and Statistical Manual of Mental Disorders version four (DSM-IV) criteria (2), that is, all of the patients had to have one or both of the two main symptoms (depressed mood, loss of interest or pleasure) that had lasted for more than two weeks.

- 1. Head motion control

The intrinsic BOLD signal contaminated by non-neuronal physiological processes and head motion is the major obstacle in the analysis of RS-fMRI data, and we performed systematic tactics to deal with these factors. First, the T1-weighted images and the T2-weighted FLAIR images were used to remove obvious gross brain structure or signals abnormalities subjects by visual inspection, and no subjects were removed. Second, the Friston 24 head motion parameters were regressed out, including six head-motion parameters (three translational and three rotational), six head-motion parameters one timepoint before, and the 12 corresponding squared items. Third, we regressed nuisance signals such as white matter (WM), cerebrospinal fluid (CSF), and also global signal. fourth, we also deal with the volume-to-volume head motion, also called framewise displacement (FDs). Using mean FDpower > 0.2mm as a threshold (not FDJenk), the maximal scrubbings volumes were counted in our study, and One-way ANOVA and post-hoc t-test were performed to explore whether these head parameters exist significant difference among the three groups. The results showed that the maximal scrubbings volumes were 27 volumes (27/230 = 11.73%) in our study. No significant difference in scrubbings volumes and the mean FDpower among the three groups was observed (scrubbing volumes: 14.82 ± 8.55, 16.02 ± 7.36, 15.34 ± 8.30; mean FDpower: 0.1016 ± 0.0588, 0.0993 ± 0.0599, 0.0909 ± 0.0541; F = 0.233, P = 0.7920; F = 0.4190, P = 0.6580). Finally, a Pearson correlation analysis was performed between the mean FDpower values and the ReHo values of these ROIs in depressed ET patients, and no significant correlation was observed, and we also used the mean FDpower values as a covariate in the correlation analysis between ReHo values of ROIs and HDRS-17 scores in depressed ET patients.

1. Supplementary Figures

**Figure S1. The flow chart of imaging data preprocessing.**


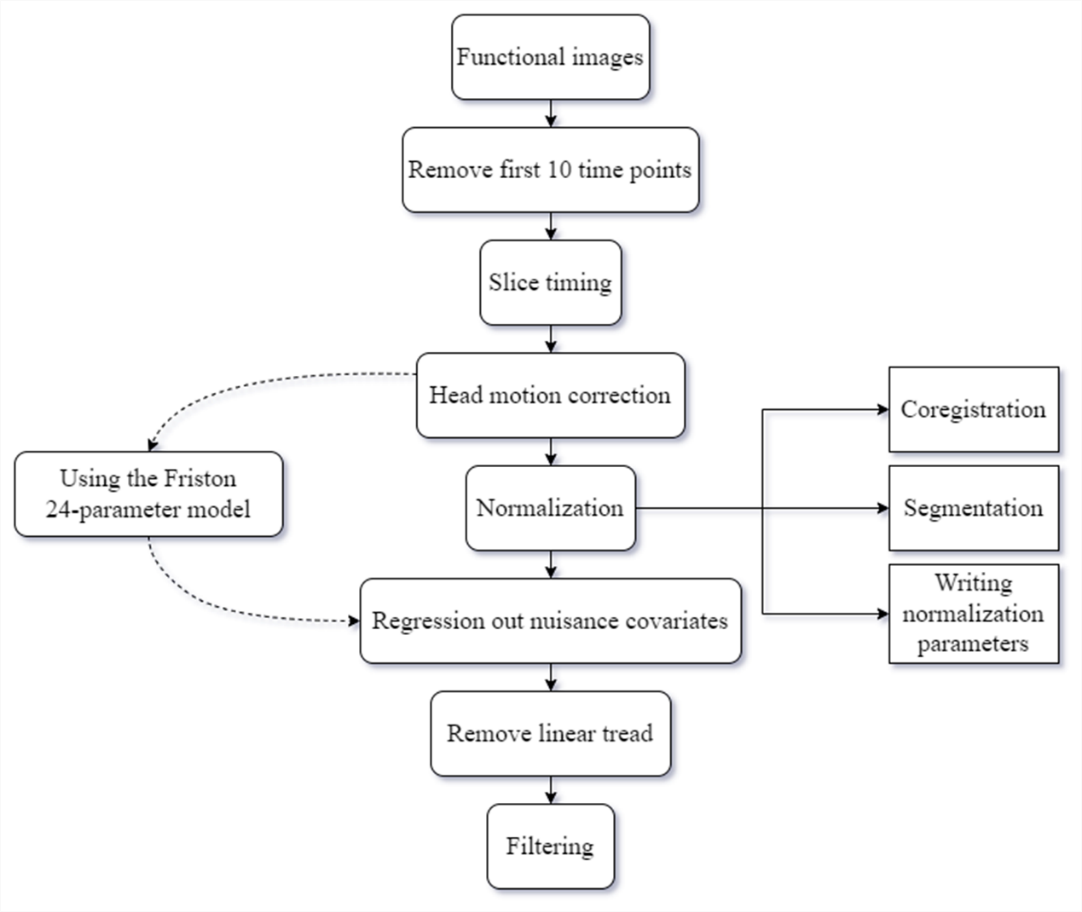


**Figure S2. The confusion matrix of the multiclass Gaussian Process Classification (MGPC) algorithm.** DET: depressed essential tremor, ET: non-depressed essential tremor, HCs: healthy controls.


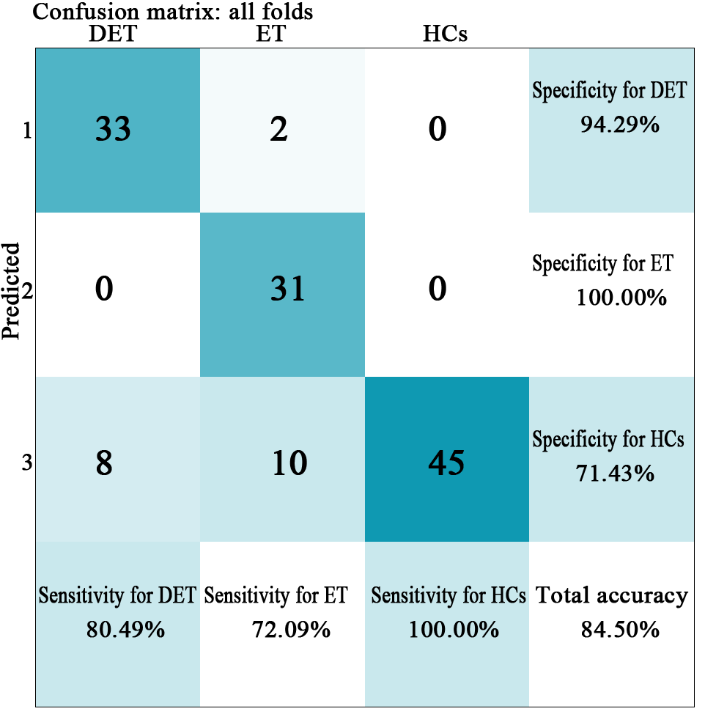


**Figure S3. The confusion matrix of the Simple Multiple Kernel Learning (Simple-MKL) algorithm.** DET: depressed essential tremor, ET: non-depressed essential tremor, HCs: healthy controls.


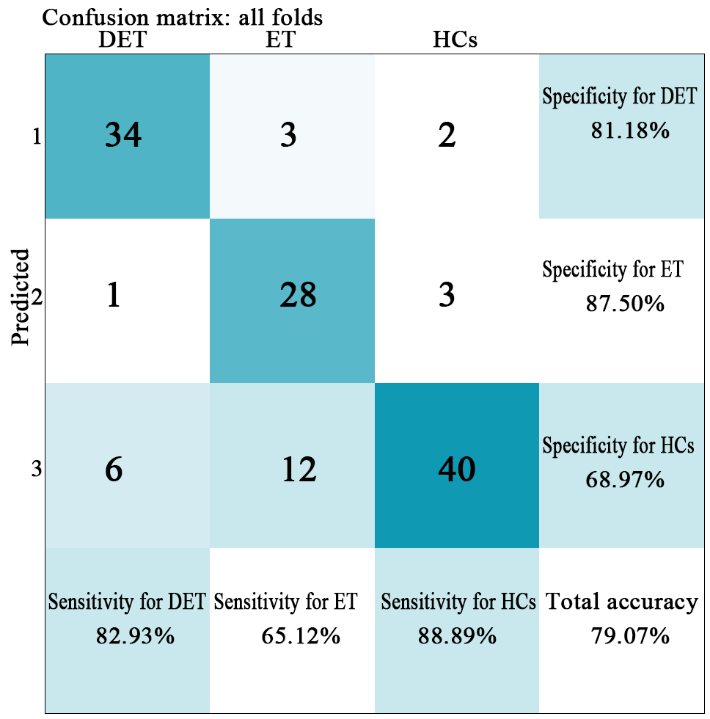


**Figure S4. The confusion matrix of the binary Gaussian Process classifier (BGPC) algorithm.** DET: depressed essential tremor, ET: non-depressed essential tremor, HCs: healthy controls.


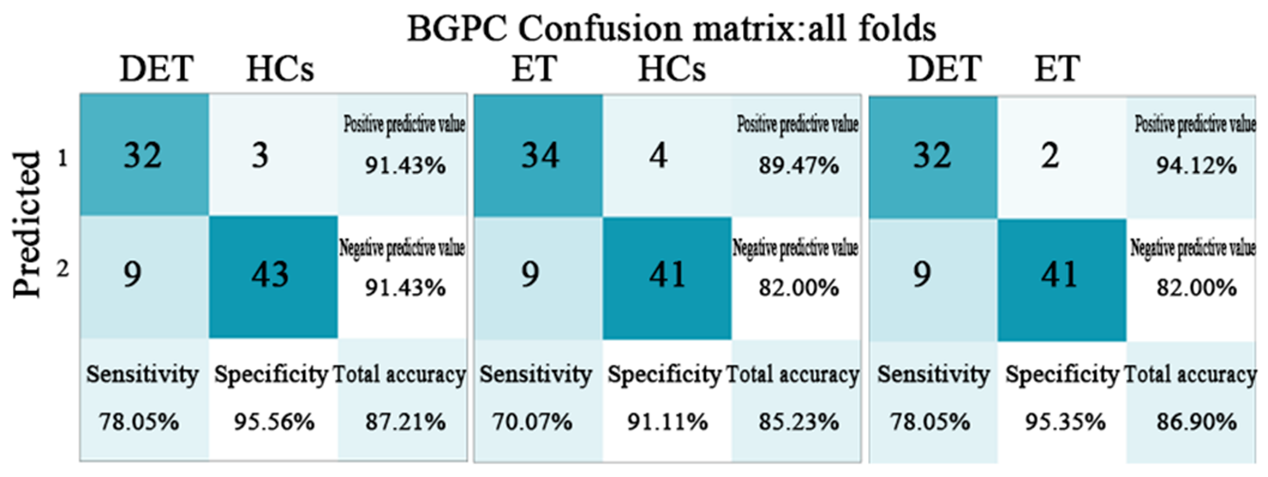


1. Supplementary table

**Table S1. The brain regions and peak MNI coordinates of significant discriminative features in classification of depressed ET *vs* HCs, non-depressed ET *vs* HCs, depressed ET *vs* non-depressed ET.**

| Brain region | Size (voxels) | *W*-value | MNI coordinates | | |
| --- | --- | --- | --- | --- | --- |
|  |  |  | *x* | *y* | *z* |
| **Depressed ET *vs* HCs** | | | | | |
| Cluster 1: cluster size: 109 voxels, peak MNI: -21 -70 43, peak *W*-value: 0.18 | | | | | |
| Left superior parietal lobule | 98 | 0.18 | -21 | -70 | 43 |
| Cluster 2: cluster size: 131 voxels, peak MNI: 46 -1 47, peak *W*-value: 0.15 | | | | | |
| Right precentral cortex | 124 | 0.15 | 46 | -1 | 47 |
| Cluster 3: cluster size: 137 voxels, peak MNI: -52 0 47, peak *W*-value: 0.14 | | | | | |
| Left precentral cortex | 129 | 0.14 | -52 | 0 | 47 |
| Cluster 4: cluster size: 167 voxels, peak MNI: -2 -14 51, peak *W*-value: 0.13 | | | | | |
| Left supplementary motor cortex | 101 | 0.13 | -2 | -14 | 51 |
| Right supplementary motor cortex | 57 | 0.09 | 2 | -9 | 51 |
| Cluster 5: cluster size: 87 voxels, peak MNI: 33 -46 56, peak *W*-value: 0.11 | | | | | |
| Right inferior parietal lobule | 81 | 0.11 | 33 | -46 | 56 |
| Cluster 6: cluster size: 157 voxels, peak MNI: -7 34 20, peak *W*-value: 0.09 | | | | | |
| Left anterior cingulum cortex | 109 | 0.09 | -7 | 34 | 20 |
| Right anterior cingulum cortex | 41 | 0.05 | 1 | 37 | 20 |
| Cluster 7: cluster size: 121 voxels, peak MNI: -45 42 20, peak *W*-value: 0.07 | | | | | |
| Left middle prefrontal gyrus | 115 | 0.07 | -45 | 42 | 20 |
| Cluster 8: cluster size: 131 voxels, peak MNI: -21 53 20, peak *W*-value: 0.06 | | | | | |
| Left superior prefrontal gyrus | 121 | 0.06 | -21 | 53 | 30 |
| Cluster 9: cluster size: 197 voxels, peak MNI: 23 53 26, peak *W*-value: 0.06 | | | | | |
| Right middle prefrontal gyrus | 93 | 0.06 | 23 | 53 | 26 |
| Right superior prefrontal gyrus | 90 | 0.05 | 21 | 55 | 18 |
| Cluster 10: cluster size: 117 voxels, peak MNI: -62 -12 7, peak *W*-value: 0.05 | | | | | |
| Left superior temporal gyrus | 96 | 0.05 | -62 | -12 | 7 |
| Cluster 11: cluster size: 107 voxels, peak MNI: 57 -27 7, peak *W*-value: 0.04 | | | | | |
| Right superior temporal gyrus | 90 | 0.04 | 57 | -27 | 7 |
| Cluster 12: cluster size: 73 voxels, peak MNI: 6 -75 22, peak *W*-value: 0.03 | | | | | |
| Right cuneus | 64 | 0.03 | 6 | -75 | 22 |
| Cluster 13: cluster size: 101 voxels, peak MNI: -3 -57 -44, peak *W*-value: -0.22 | | | | | |
| Left cerebellum IX | 93 | -0.22 | -3 | -57 | -44 |
| Cluster 14: cluster size: 89 voxels, peak MNI: 7 -58 -44, peak *W*-value: -0.21 | | | | | |
| Right cerebellum IX | 73 | -0.21 | 7 | -58 | -44 |
| Cluster 15: cluster size: 101 voxels, peak MNI: 9 -65 -33, peak *W*-value: -0.15 | | | | | |
| Right cerebellum VIII | 89 | -0.15 | 9 | -65 | -33 |
| Cluster 16: cluster size: 87 voxels, peak MNI: -13 -59 -39, peak *W*-value: -0.13 | | | | | |
| Left cerebellum VIII | 77 | -0.13 | -13 | -59 | -39 |
| Cluster 17: cluster size: 135 voxels, peak MNI: 25 -67 -25, peak *W*-value: -0.11 | | | | | |
| Right cerebellum VI | 69 | -0.11 | 25 | -67 | -25 |
| Right cerebellum crus 1 | 59 | -0.07 | 23 | -71 | -33 |
| Cluster 18: cluster size: 101 voxels, peak MNI: -20 -72 -28, peak *W*-value: -0.06 | | | | | |
| Left cerebellum crus 1 | 93 | -0.06 | -20 | -72 | -28 |
| Cluster 19: cluster size: 167 voxels, peak MNI: -10 -53 -20, peak *W*-value: -0.05 | | | | | |
| Left cerebellum IV~V | 87 | -0.05 | -10 | -53 | -20 |
| Left cerebellum VI | 73 | -0.04 | -18 | -56 | -20 |
| Cluster 20: cluster size: 113 voxels, peak MNI: 12 -52 -20, peak *W*-value: -0.04 | | | | | |
| Right cerebellum IV~V | 95 | -0.05 | 12 | -52 | -20 |
| **Non-depressed ET *vs* HCs** | | | | | |
| Cluster 1: cluster size: 143 voxels, peak MNI: 48 -1 49, peak *W*-value: 0.15 | | | | | |
| Left precentral cortex | 135 | 0.15 | 48 | -1 | 49 |
| Cluster 2: cluster size: 139 voxels, peak MNI: -45 -3 49, peak *W*-value: 0.14 | | | | | |
| Right precentral cortex | 127 | 0.14 | -45 | -3 | 49 |
| Cluster 3: cluster size: 135 voxels, peak MNI: -22 -71 42, peak *W*-value: 0.14 | | | | | |
| Left superior parietal lobule | 123 | 0.14 | -22 | -71 | 42 |
| Cluster 4: cluster size: 147 voxels, peak MNI: 35 -48 56, peak *W*-value: 0.14 | | | | | |
| Right inferior parietal lobule | 133 | 0.14 | 35 | -48 | 56 |
| Cluster 5: cluster size: 211voxels, peak MNI: -2 -13 55, peak *W*-value: 0.13 | | | | | |
| Left supplementary motor cortex | 107 | 0.13 | -2 | -13 | 55 |
| Right supplementary motor cortex | 89 | 0.11 | 1 | -9 | 55 |
| Cluster 6: cluster size: 113 voxels, peak MNI: -62 -13 6, peak *W*-value: 0.11 | | | | | |
| Left superior temporal gyrus | 89 | 0.11 | -62 | -13 | 6 |
| Cluster 7: cluster size: 69 voxels, peak MNI: 55 -27 6, peak *W*-value: 0.10 | | | | | |
| Right superior temporal gyrus | 61 | 0.10 | 55 | -27 | 6 |
| Cluster 8: cluster size: 63 voxels, peak MNI: 7 -79 24, peak *W*-value: 0.08 | | | | | |
| Right cuneus | 53 | 0.08 | 7 | -79 | 24 |
| Cluster 9: cluster size: 159 voxels, peak MNI: -2 37 22, peak *W*-value: 0.05 | | | | | |
| Left anterior cingulum cortex | 89 | 0.07 | -2 | 37 | 22 |
| Right anterior cingulum cortex | 39 | 0.03 | 3 | 36 | 22 |
| Cluster 10: cluster size: 104 voxels, peak MNI: -42 42 22, peak *W*-value: 0.04 | | | | | |
| Left middle prefrontal gyrus | 91 | 0.05 | -42 | 42 | 22 |
| Cluster 11: cluster size: 76 voxels, peak MNI: -18 51 24, peak *W*-value: 0.03 | | | | | |
| Left superior prefrontal gyrus | 67 | 0.03 | -18 | 51 | 24 |
| Cluster 12: cluster size: 111 voxels, peak MNI: 24 51 26, peak *W*-value: 0.03 | | | | | |
| Right middle prefrontal gyrus | 59 | 0.03 | 24 | 51 | 26 |
| Right superior prefrontal gyrus | 37 | 0.03 | 20 | 51 | 21 |
| Cluster 13: cluster size:123voxels, peak MNI: 9 -66 -33, peak *W*-value: -0.18 | | | | | |
| Right cerebellum VIII | 117 | -0.18 | 9 | -66 | -33 |
| Cluster 14: cluster size:117 voxels, peak MNI: -13 -59 -40, peak *W*-value: -0.15 | | | | | |
| Left cerebellum VIII | 97 | -0.15 | -13 | -59 | -40 |
| Cluster 15: cluster size:274 voxels, peak MNI: 19 -66 -28, peak *W*-value: -0.10 | | | | | |
| Left cerebellum VI | 115 | -0.10 | -18 | -57 | -20 |
| Left cerebellum IV~V | 79 | -0.07 | -14 | -53 | -20 |
| Left cerebellum crus 1 | 59 | -0.04 | -21 | -72 | -28 |
| Cluster 16: cluster size:171 voxels, peak MNI: 19 -66 -28, peak *W*-value: -0.06 | | | | | |
| Right cerebellum VI | 114 | -0.06 | 28 | -66 | -28 |
| Right cerebellum crus 1 | 47 | -0.03 | 23 | -67 | -35 |
| Cluster 17: cluster size:91 voxels, peak MNI: 13 -49 -20, peak *W*-value: -0.04 | | | | | |
| Right cerebellum IV~V | 77 | -0.04 | 13 | -49 | -20 |
| **Depressed ET *vs* non-depressed ET** | | | | | |
| Cluster 1: cluster size: 53 voxels, peak MNI: -3 38 25, peak *W*-value: 0.05 | | | | | |
| Left anterior cingulum cortex | 47 | 0.05 | -3 | 38 | 25 |
| Cluster 2: cluster size: 51 voxels, peak MNI: -44 42 24, peak *W*-value: 0.03 | | | | | |
| Left middle prefrontal gyrus | 37 | 0.03 | -44 | 42 | 24 |
| Cluster 3: cluster size:43 voxels, peak MNI: -19 -68 -24, peak *W*-value: 0.02 | | | | | |
| Left superior prefrontal gyrus | 33 | 0.02 | -20 | 54 | 22 |
| Cluster 4: cluster size: 57 voxels, peak MNI: 23 55 31, peak *W*-value: 0.02 | | | | | |
| Right superior prefrontal gyrus | 49 | 0.02 | 23 | 55 | 31 |
| Cluster 5: cluster size: 101 voxels, peak MNI: -8 -57 -41, peak *W*-value: -0.14 | | | | | |
| Left cerebellum IX | 83 | -0.14 | -8 | -57 | -41 |
| Cluster 6: cluster size: 103 voxels, peak MNI: 8 -58 -44, peak *W*-value: -0.10 | | | | | |
| Right cerebellum IX | 75 | -0.10 | 8 | -58 | -44 |

**References**

1. Bhatia KP, Bain P, Bajaj N, Elble RJ, Hallett M, Louis ED, et al. Consensus Statement on the classification of tremors. from the task force on tremor of the International Parkinson and Movement Disorder Society. *Mov Disord.* (2018) 33:75-87. doi: 10.1002/mds.27121

2. Cooper JE. On the publication of the Diagnostic and Statistical Manual of Mental Disorders: Fourth Edition (DSM-IV). *Br J Psychiatry.* (1995) 166:4-8. doi: 10.1192/bjp.166.1.4
